# *Isatis tinctoria* L. Leaf Extract Inhibits Replicative Senescence in Dermal Fibroblasts by Regulating mTOR-NF-κB-SASP Signaling

**DOI:** 10.3390/nu14091979

**Published:** 2022-05-09

**Authors:** Jieun Woo, Seoungwoo Shin, Hyanggi Ji, Dehun Ryu, Eunae Cho, Youngseok Kim, Junoh Kim, Deokhoon Park, Eunsun Jung

**Affiliations:** 1 BioSpectrum Life Science Institute, 767, Sinsu-ro, Yongin-si 16827, Korea; biotw@biospectrum.com (J.W.); biost@biospectrum.com (S.S.); biocr@biospectrum.com (H.J.); biosc@biospectrum.com (D.R.); biozr@biospectrum.com (E.C.); pdh@biospectrum.com (D.P.); 2 Shinsegae International Technology Innovation Center, 449, Dosan-daero, Seoul 06015, Korea; yskim23@sikorea.co.kr (Y.K.); junohkim@sikorea.co.kr (J.K.)

**Keywords:** *Isatis tinctoria* L., senomorphics, replicative senescence, senescence-associated secretory phenotype (SASP), anti-aging, mTOR pathway

## Abstract

Senescent fibroblasts progressively deteriorate the functional properties of skin tissue. Senescent cells secrete senescence-associated secretory phenotype (SASP) factor, which causes the aging of surrounding non-senescent cells and accelerates aging in the individuals. Recent findings suggested the senomorphic targeting of the SASP regulation as a new generation of effective therapeutics. We investigated whether *Isatis tinctoria* L. leaf extract (ITE) inhibited senescence biomarkers *p53*, *p21^CDKN1A^*, and *p16^INK4A^* gene expression, and SASP secretions by inhibiting cellular senescence in the replicative senescent human dermal fibroblast (RS-HDF). ITE has been demonstrated to inhibit the secretion of SASP factors in several senomorphic types by regulating the MAPK/NF-κB pathway via its inhibitory effect on mTOR. ITE suppressed the inflammatory response by inhibiting mTOR, MAPK, and IκBα phosphorylation, and blocking the nuclear translocation of NF-κB. In addition, we observed that autophagy pathway was related to inhibitory effect of ITE on cellular senescence. From these results, we concluded that ITE can prevent and restore senescence by blocking the activation and secretion of senescence-related factors generated from RS-HDFs through mTOR-NF-κB regulation.

## 1. Introduction

As a characteristic of cellular senescence, the phenomenon of cell division cessation is caused by various damaging stimuli, such as oxidative stress, irradiation, telomere dysfunction, and oncogenic stress. As a method for identifying senescent cells, an increase in senescence-associated β-galactosidase (SA-β-gal) activity is considered a representative standard. Other than increased SA-β-gal activity, the characteristics of senescent cells include an enlarged, flattened, and irregular cell morphology, activation of *p16^INK4A^* expression, reduced lamin B1 expression, high mobility group box 1 (HMGB1) translocated into the cytoplasm and released extracellularly, and the secretion of senescence-associated secretory phenotype (SASP) factors such as inflammatory cytokines and metalloproteinases [1].

We verified changes in the chronic senescence environment by inducing replicative senescence in cultured cells by repeated passages in various senescent models. Chronic senescence can result from long-term unexpected damage, and it is often associated with harmful processes such as aging. In chronic senescence, senescent cells accumulate in organs and mediate cellular dysfunction via SASP release. Recent reports have shown that the function of senescent cells in vivo was significantly affected by the effects of SASP on the surrounding environment and the associated immune responses [2]. SASP factors that enhance and induce secondary senescence of surrounding cells contribute to aging and tissue degeneration, affecting them in an autocrine and paracrine manner. For treating aging and age-related diseases, regulation of SASP factors or inhibition of the SASP-mediated detrimental cycle are important strategies [3].

Small molecules that aim to inhibit all or some senescent cells characteristics through SASP blockade are termed senomorphics and indirectly interfere with the senescence phenomenon, acting as SASP inhibitors without cell death [4]. Free radical scavengers and various inhibitors including Janus kinase (JAK), IκB kinase (IKK), and nuclear factor (NF)-κB have been reported as senomorphics of several class. Also, rapamycin inhibiting the mammalian target of rapamycin (mTOR) acts as a senomorphic by reducing the SASP to suppress senescence [5].

The mTOR pathway regulates metabolism and aging in response to nutrition and growth factors and is also involved in the acquisition of the senescent phenotype [6]. The role of mTOR in inducing senescence-associated phenotypes including the SASP has been comprehensively established, and tissue changes such as fibrosis and cellular transformation have been closely associated with the inflammatory environment mediated by the SASP. mTOR also acts as a negative regulator of autophagy, and rapamycin induces autophagy through the inhibition of mTOR [7]. Therapeutic approaches aimed at modulating the senescent phenotypes could more safely mitigate the deleterious effects of senescent cells in vitro. In this study, we evaluated the role of *Isatis tinctoria* L. leaf extract (ITE) as a senomorphic agent in the treatment of skin aging.

*Isatis tinctoria* L., commonly known as woad, is a biennial or short-lived perennial herb native to Central Asia. It has been appreciated for its anti-inflammatory effects, is widely utilized for medicinal purposes in traditional Chinese medicine (TCM), and has been recognized as a pharmacopoeial plant in Europe over the past decades [8]. People of Sicily in Italy consume the flower buds as an ingredient in salads and omelets [9], while in China, the roots and leaves are consumed as herbal tea to prevent sore throat and hepatitis. In Korea, young leaves have been used as food. According to the Korean traditional medicine book, Donguibogam, *I. tinctoria* is recorded as an herbal medicine for treating high fever and stomatitis. The bioactivities of *I. tinctoria* in anti-oxidant, anti-inflammatory [10], anti-viral, and anti-tumor effects have been recently reported [9], and clinical study showed the protective effect of ITE on dermatitis due to sodium lauryl sulfate (SLS) contact and erythema caused by UVB irradiation [11]. However, the effect of *Isatis tinctoria* L. on skin aging and the involvement of this pathway in senescence regulation has not yet been investigated. Therefore, we investigated whether ITE suppressed the dynamic fluctuation of mTOR/MAPK/NF-κB activation, which induced SASP secretions in replicative senescent human dermal fibroblasts (RS-HDFs), thereby reinforcing and amplifying autophagy.

## 2. Materials and Methods

### 2.1. Plant Preparation and Extraction

The dried leaves of *Isatis tinctoria* L. were purchased from a local herbal shop (Samhong geonjae yag-eobsa, Seoul, Korea). To prepare the ethanolic extract, 2.46 kg of the dried leaves were extracted with 40 L of 70% ethanol (*w*/*w*) at 80 °C for 3 h, and the leaves were removed using filter paper (Advantec, No. 131 qualitative filter paper (3 μm)). The extract was evaporated for removing alcoholic part via a rotary vacuum evaporator (EYELA, Tokyo, Japan) and was lyophilized. The dark brown powder (0.672 kg) was obtained and dissolved in DMSO for further experiments.

### 2.2. Cell Culture

Human dermal fibroblasts (HDFs; PCS-201-010™) cultured in Dulbecco’s modified Eagle’s medium (DMEM) with 10% fetal bovine serum (FBS; Welgene, Daegu, Korea) and 100 IU/50 μg/mL penicillin/streptomycin in a humidified atmosphere containing 5% CO_2_ at 37 °C. Young HDFs were used for the experiments from passages 8 to 10. RS-HDFs were induced by continual passaging of the cells in tissue culture to acquire the senescence phenotype after 40 passages.

### 2.3. Lactate Dehydrogenase Assay

The LDH assay measured lactate dehydrogenase (LDH) released from damaged cells to determine cytotoxicity. HDFs (2 × 10^4^ cells/24-well plate) were treated with the ITE for 72 h. After incubation, the assay was conducted using an LDH Cytotoxicity WST Assay kit (Enzo Life Sciences, Farmingdale, NY, USA). Lysis buffer was used for the measurement of total cellular LDH [12].

### 2.4. SA-β-Gal Staining Assay

HDFs (1 × 10^5^ cells/6-well plate) were treated with the ITE for 72 h. For flow cytometry, the cells were stained with a SPiDER-βGal (Dojindo, Kumamoto, Japan). SA-β-gal was used as an indicator of cellular senescence. The cells were incubated with the staining solution for 15 min at 37 °C after adding and analyzed by flow cytometry.

### 2.5. Luminex Cytokine Assay

Supernatants were collected 72 hr after treatment, and human interleukin (IL)-1α, IL-6, IL-8, and tumor necrosis factor (TNF)-α were detected using the Luminex kit (R&D system Inc., Minneapolis, MN, USA) according to the manufacturer’s protocol. The plates were read using the MAGPIX^®^. The protein concentrations were analyzed using Luminex xPONENT® software (ver. 4.2; Luminex Corporation, Austin, TX, USA).

### 2.6. Immunofluorescence Staining

HDFs (2 × 10^4^ cells/24-well plate) treated with the ITE for 24 hr. After incubation, cells were fixed with 4% formaldehyde at room temperature for 15 min, followed by blocking with 5% bovine serum albumin (BSA) including 0.3% Triton X-100 (Biosesang, Seongnam, Korea) in phosphate-buffered saline (PBS) for 1 h. Anti–p65 antibodies (1:50 dilution; Invitrogen, Carlsbad, CA, USA) with 5% BSA including 0.3% Triton X-100 in PBS were added to each well, followed by incubation overnight at 4 °C. The cells were washed twice with PBS and stained nucleic acid with Hoechst 33342 (1:10,000 dilution; Invitrogen, Carlsbad, CA, USA) at room temperature for 10 min. Subsequently, fluorescence was analyzed by fluorescence microscopy (Life Technologies GmbH, Darmstadt, Germany)

### 2.7. Quantitative Polymerase Chain Reaction (qRT-PCR)

The mRNA of the HDFs was extracted by using TRIzol™ (Invitrogen, CA, USA) according to the manufacturer’s protocol. The mRNA sample (1 mg) was reverse transcribed to cDNA by using amfiRivert cDNA Synthesis Platinum master Mix (GenDEPOT, Barker, TX, USA). Relative gene expression was analyzed with the 7500 Real-Time PCR system (Applied Biosystems, Foster City, CA, USA). Reaction mixture with each primer (Bioneer, Daejeon, Korea) and AMPIGENE^®^ qPCR Green Mix (Enzo Life Sciences, Farmingdale, NY, USA) were applied to 40 cycles of RT-PCR (95 °C for 15 s, 60 °C for 60 s, and 95 °C for 10 min). The relative gene expression against the *GAPDH* gene was evaluated based on the comparative or ΔΔCt method. Table 1 lists the primer sequences used in this study.

### 2.8. Western Blots

Total proteins were separated with a Bio-rad electrophoresis system (Bio-rad, Hercules, CA, USA) and transferred to polyvinylidene difluoride (PVDF) membranes. Immunoblotting was performed using the primary antibodies, anti-mTOR (Novus Biologicals, Littleton, CO, USA), anti-histone H3 asymmetric di-methyl R17 (Abcam, Cambridge, UK), and anti-human SIRT1 (LSBio, Seattle, WA, USA). Phospho-IκBα (Ser32), IκBα, phospho-p44/42 MAPK (Erk1/2) (Thr202/Tyr204), phospho-SAPK/JNK (Thr183/Tyr185), phospho-mTOR (Ser2448), and phospho-S6 ribosomal protein (Ser235/236) antibodies were also obtained from Cell Signaling Technology (Beverly, MA, USA). MMP-1 and GAPDH were used and purchased from Santa Cruz Biotechnology, Inc. (Dallas, TX, USA). Primary antibodies were used diluted 1:500–1000 in 5% BSA of tris-buffered saline with 0.1% Tween 20 (TBS/T). Scanning densitometric values of bands were analyzed using ImageJ, software version 1.52a (National Institutes of Health, Rockville, MD, USA).

### 2.9. Liquid Chromatography with Tandem Mass Spectrometry (LC-MS/MS)

The profiling analysis was carried out on the LC-MS/MS system, which consisted of an Ultimate 3000 (Thermo Scientific, Waltham, MA, USA) and a Triple TOF™ 5600 (AB SCIEX, Framingham, MA, USA). Chromatographic separation was performed with a Waters Cortex C18 column (2.1 mm × 150 mm, 1.6 μm) with a mobile phase of 0.1% formic acid in water (A) and 0.1% formic acid in acetonitrile (B) at 45° C. The mobile phase gradient was as follows: from 0 to 1 min, 5% B; 1 to 30 min, 5% to 30% B; 30 to 50 min, and 30 to 100% B. The flow rate was 0.3 mL/min. Mass spectrometry was performed based on information-dependent acquisition (IDA) scanning with electrospray ionization (ESI) in the negative and positive ion modes. For the MS/MS system, nitrogen gas was used in fragmentation at 500 °C and 50 psi, and the condition of positive mode was as follows: ion spray voltage, 5.5 kV; declustering potential (DP), 60 V; collision energy (CE), 10 eV, and the condition at negative mode was as follows: ion spray voltage, 4.5 kV; DP, −60 V; CE, −10 eV. The mass scan range was from 100 to 2000 m/z.

### 2.10. Statistical Analysis

All values are expressed as the mean ± SD (standard deviation). All experiments were conducted in triplicate, and the statistical significance of all data was evaluated by the Student’s t-test using Statview software (ver. 5.0; Abacus Concepts, Berkeley, CA, USA). A *p*-value of <0.05 indicated statistical significance.

## 3. Results

### 3.1. ITE Inhibits Replicative Senescence and Oxidative Stress

**Figure 1:**
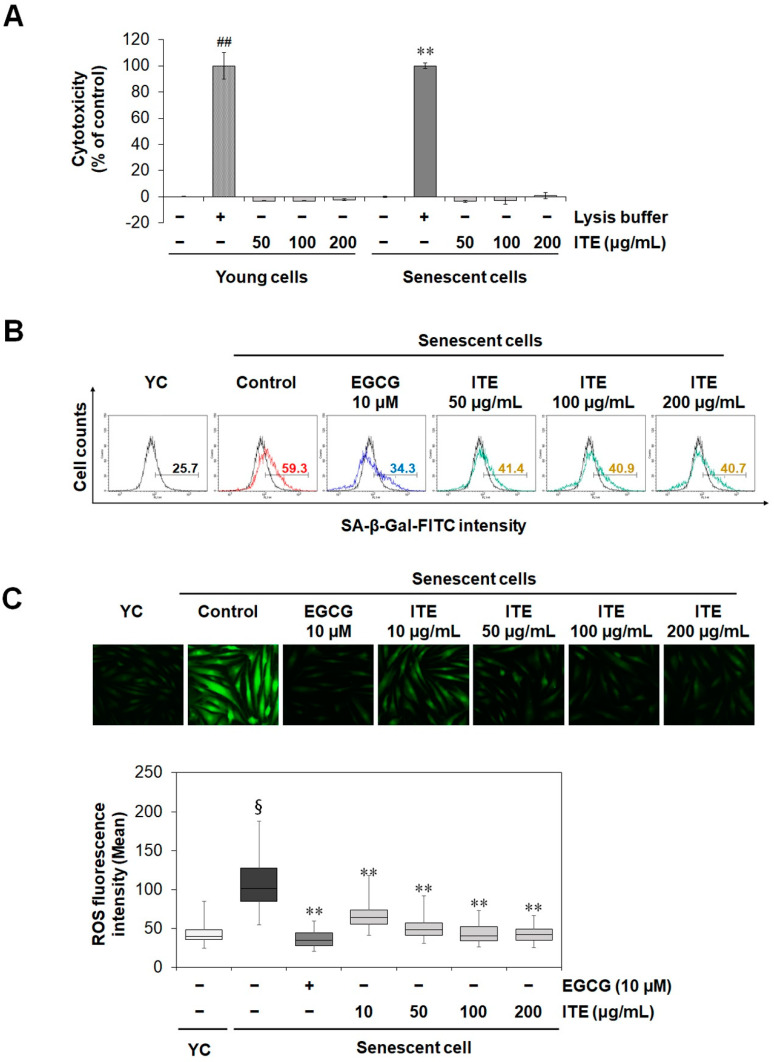
Inhibitory effect of ITE on SA-β-gal activity and oxidative stress in RS-HDFs. (**A**) The cytotoxicity of ITE in HDFs was determined by LDH assays. Lysis buffer was used as a positive control. (**B**) Representative flow cytometry plots are shown. Values inside the plots quantified the percentage of positively stained cells (SA-β-gal FITC-positive) detected by flow cytometry. (**C**) Images of HDF fluorescence induced by ROS are shown as DCF fluorescence emitting green fluorescence. Representative immunofluorescence images and fluorescence intensity analysis. The data are presented as the mean ± standard error of the mean (SEM) of three independent assays. ## *p* < 0.01 or § *p* < 0.01 compared to young HDF controls, ** *p* < 0.01 compared to RS-HDF controls. YC, young cell controls.

**Figure 2:**
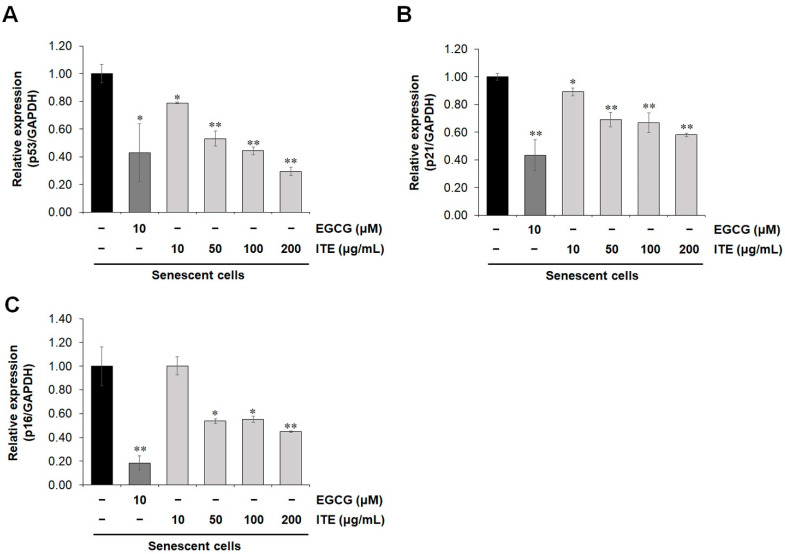
Inhibitory effect of ITE on tumor suppressors in RS-HDFs. qRT–PCR for *p53* (**A**), *p21^CDKN1A^* (**B**), *p16^INK4A^* (**C**) mRNA expression. The data are presented as the mean ± SEM of three independent assays. * *p* < 0.05; ** *p* < 0.01 compared to RS-HDF control.

Cellular senescence occurs through many pathways such as replicative senescence, oncogene-induced senescence, and stress-induced senescence. Among them, the replicative senescent human fibroblast is a widely used cellular model for human aging. Telomere erosion was reported to contribute to the development of replicative senescence [13]. To induce replicative senescence, HDF cells were allowed to grow for more than 40 passages. Cell proliferation rapidly decreased, and senescence indicators increased. To investigate the anti-aging role of ITE on RS-HDFs, SA-β-gal staining was performed at a non-cytotoxic concentration (Figure 1A). As shown in Figure 1B, the number of SA-β-gal-positive RS-HDFs was significantly increased (59.3%) compared to young HDFs (25.7%). However, ITE decreased the stained SA-β-gal positive cells by 40.7% at 200 μg/mL. Epigallocatechin gallate (EGCG) is a positive control anti-aging agent and also reduced SA-β-gal-positive cells by 34.3%. Reactive oxygen species (ROS) is a major inducer of replicative senescence and is increased in replicative senescent cells [14,15]. We observed that the increased oxidative stress in RS-HDFs was suppressed by ITE treatment (Figure 1C). The activation of tumor suppressors including *p53/p21^WAF1^* and *p16^INK4A^* is considered a hallmark of senescence [16]. Consistent with the SA-β-gal and ROS results, the mRNA expression of *p53*, *p21^CDKN1A^*, and *p16^INK4A^* was dose-dependently decreased in RS-HDFs treated with ITE (0.30, 0.58 and 0.45-fold reductions, respectively, at a concentration of 200 μg/mL) (Figure 2). These data suggest that ITE induced decreases in senescence mediated by ROS and the activation of the *p53-p21^CIP1^* pathway, as well as *p16^INK4A^*, in RS-HDFs.

### 3.2. ITE Shows a Senomorphic Effect by Inhibiting Senescence-Associated Secretory Phenotype (SASP) Factor Production in Senescent Fibroblasts

**Figure 3:**
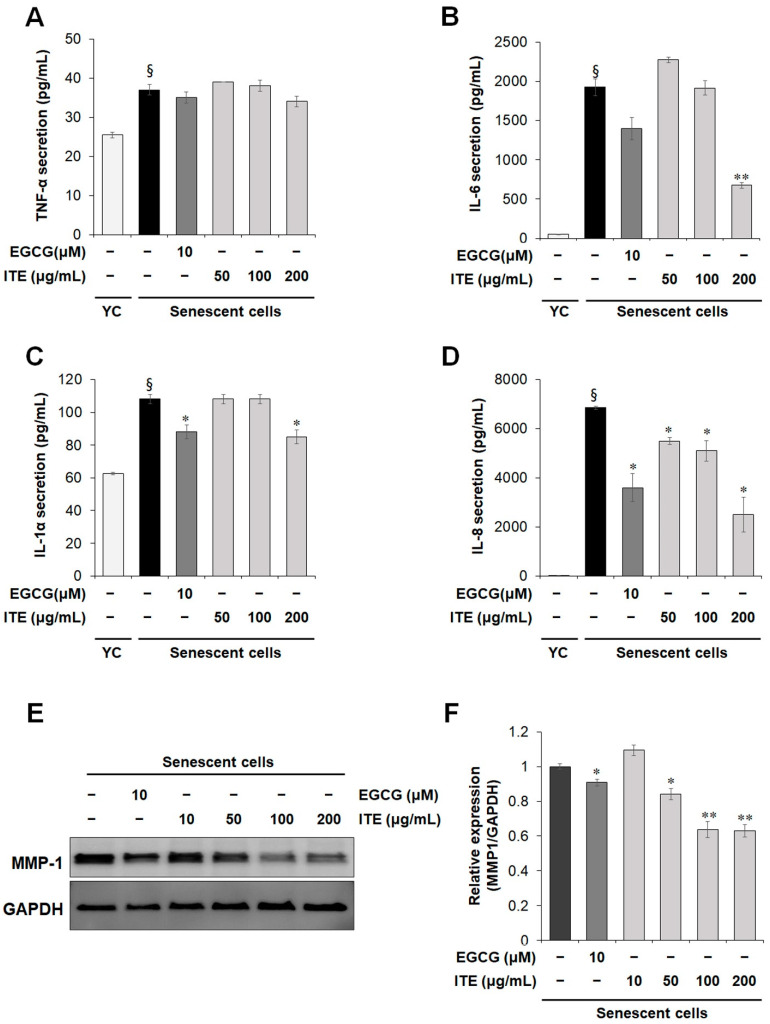
Inhibitory effect of ITE on SASP expression in RS-HDFs. The secretion of TNF-α (**A**), IL-6 (**B**), IL-1α (**C**), and IL-8 (**D**) in the culture supernatant was measured using a Luminex kit. The expression level of MMP-1 was determined by Western blots. (**E**) Densitometric ratio graph of MMP-1/GAPDH (**F**). The data are presented as the mean ± SEM of three independent assays. § *p* < 0.01 compared to young HDF controls, * *p* < 0.05; ** *p* < 0.01 compared to RS-HDF controls.

As it is widely known that the expression and secretion of SASP such as inflammatory cytokines and metalloproteinases (MMPs) are increased in senescent cells. This finding was confirmed by Luminex as shown in Figure 3A–D where the increase in RS-HDF SASP secretions, TNF-α, IL-1α, IL-6, and IL-8 was lower following ITE treatment. IL-6 and IL-8 were significantly increased in RS-HDFs by 40 times and 600 times, respectively, compared to young HDFs. After treatment with 200 μg/mL ITE, they were decreased by more than 70% compared to control RS-HDFs. Likewise, when the expression of matrix metalloproteinase (MMP)-1, a SASP marker, was analyzed by Western blots, the expression level that was increased due to cellular senescence was decreased by ITE treatment (Figure 3E,F). These results suggest that ITE suppressed replicative senescence and can be considered senomorphic through its effect to decrease SASP secretions in RS-HDFs.

### 3.3. ITE Suppresses the Activation of MAPK (JNK and ERK), IκB, and NF-κB in RS-HDFs

**Figure 4:**
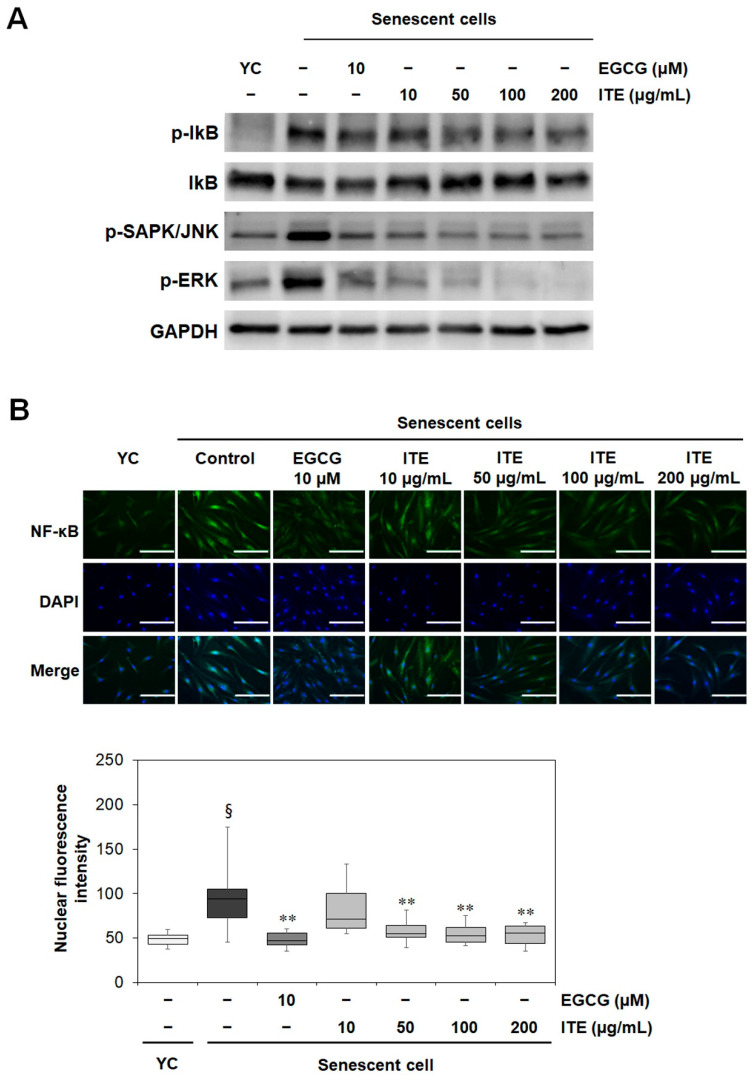
Inhibitory effect of ITE on the expression of IκBα, JNK, and ERK and NF-kB translocation to the nucleus in RS-HDFs. (**A**) Western blot was used to determine the protein expression levels. Each of the figures is representative of 3 independent experiments. GAPDH was used as a control to confirm equal protein loading. (**B**) Representative immunofluorescence image and nuclear fluorescence intensity analysis. Cells were stained with antibodies to p65 (green) and 4′,6-diamidino-2-phenylindole (DAPI; blue) and captured at ×200. The data are presented as the mean ± SEM of three independent assays. § *p* < 0.01 compared to young HDF controls, ** *p* < 0.01 compared to RS-HDF controls. YC, young cell controls.

Previous studies also reported that MAPK was activated and affected the SASP through NF-κB signaling in senescent cells [5]. In response to inflammatory stimuli, IκBα is phosphorylated and released from NF-κB. NF-κB then translocates to the nucleus and upregulates inflammation-related genes expression such as inflammatory cytokines [17]. When we confirmed the change in MAPK by Western blot analysis, the activation of JNK and ERK in the RS-HDFs was significantly reduced by EGCG and ITE treatment, and the phosphorylation of IκB was also decreased (Figure 4A). IL-6 and -8, which was significantly reduced by ITE, are known to be regulated by NF-κB [18]. Therefore, to identify the MAPK-NF-κB signals regulating the SASP such as IL-6 and IL-8, direct changes in NF-κB by ITE treatment were investigated. When the NF-κB translocation in RS-HDFs was compared by immunofluorescence (IF) staining, the translocation of NF-κB to the nucleus in RS-HDFs was decreased by EGCG and ITE treatment compared to young HDFs (Figure 4B). These results indicated that ITE abrogated the inflammatory response through NF-κB in replicative senescence.

### 3.4. ITE Suppresses SASP Secretions and Cellular Senescence by Modulating SIRT1-mTOR-MAPK-NF-κB Signaling in RS-HDFs

**Figure 5:**
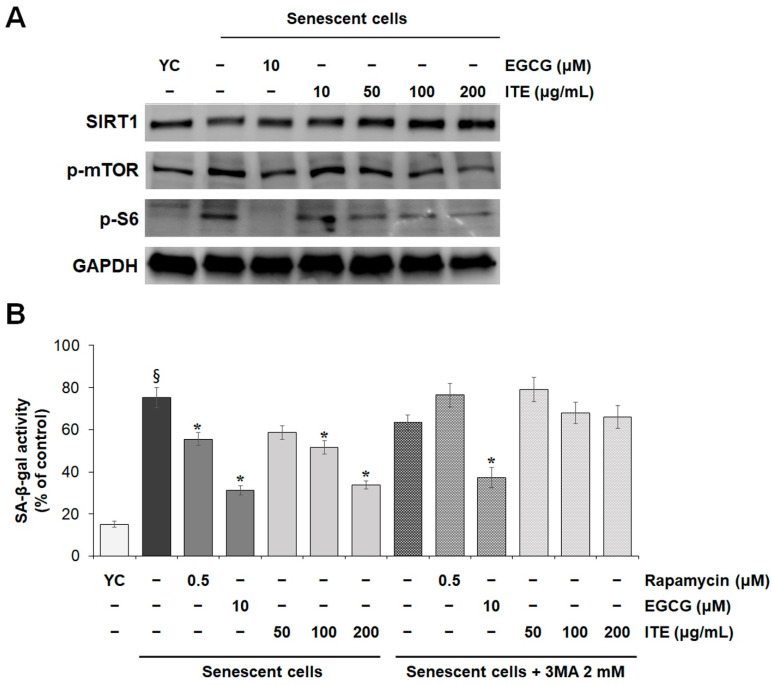
Regulatory effect of ITE on the expression of SIRT1 and mTOR in RS-HDFs. (**A**) Western blot was used to determine the protein expression levels. Each of the figures is representative of 3 independent experiments. GAPDH was used as a control to confirm equal protein loading. (**B**) Effect of simultaneous treatment with ITE and 2 mM 3MA on SA-β-gal activity in RS-HDFs. The data are presented as the mean ± SEM of three independent assays. § *p* < 0.01 compared to young HDF controls, * *p* < 0.05 compared to RS-HDF controls. YC, young cell controls.

The interaction between mTOR signaling activated during senescence, and the MAPK pathway has prompted the members of the phosphorylation cascade to be considered senomorphic targets [5]. The measurement of mTOR affecting MAPK confirmed that the increased expression level in RS-HDFs consistent with MAPK was decreased in a dose-dependent manner by ITE treatment. EGCG treatment showed a similar result. mTORC1 stimulates RNA translation by phosphorylating downstream target proteins such as p70 ribosomal S6 kinase1 (p70S6K1) and S6 ribosomal protein [19]. The phosphorylation status of the downstream substrate protein S6 decreased according to the mTOR signaling response. SIRT1 has also been reported to function through the mTOR pathway [20], and SIRT1 expression was reduced under senescent conditions compared to normal growth conditions [21]. The expression of SIRT1 was decreased in senescent HDFs compared to young HDFs, but ITE treatment significantly promoted SIRT1 expression (Figure 5A). These results suggest that ITE can be considered a modulator of SIRT1/mTOR/MAPK/NF-kB signaling, which may decrease SASP secretions.

To verify that the anti-aging effect of ITE was due to mTOR inhibition, RS-HDFs were treated with ITE or rapamycin with 2 mM 3-methyladenine (3MA), an autophagy inhibitor, and the changes were observed. As shown in Figure 5B, SA-β-gal activity was decreased by up to 55% in cells treated with 200 μg/mL ITE, 26% by rapamycin treatment, an mTOR inhibitor, and 58% by EGCG treatment. However, the anti-aging efficacy was weakened by 3MA co-treatment, whereby SA-β-gal inhibition decreased from 55% to 22% in 200 μg/mL ITE-treated cells. Also, the cellular senescence inhibitory effect was abolished when RS-HDFs were co-treated with rapamycin and 3MA, but that was maintained without a significant change in the efficacy of EGCG. These results suggest that the anti-senescence effects of ITE resulted from mTOR inhibition and autophagy.

## 4. Discussion

Aging is the result of complex biological interactions that impair biological, physical and biochemical processes by causing cumulative damage to molecules, cellular functions, and organs.

The accumulation of senescent cells can deplete stem and progenitor cell compartments necessary for tissue repair and regeneration. In addition, skin aging is closely related to the cellular aging of dermal fibroblasts. Fibroblasts release SASP factors, such as specific inflammatory cytokines and senescence factors, as cellular senescence progresses [21]. Senescent cells change their microenvironment by communicating through direct cell-cell contact, cell fusion, cytoplasmic bridge formation, extracellular vesicle (EV) signaling, and the SASP, and most of the non-autonomous effects of senescent cells are related to the SASP. Thus, senomorphics play a role in modulating the SASP, a primary mediator of the detrimental effects of senescent cells, which is important from the aspect of cellular senescence control [22]. Several methods to suppress senescence, such as senolytics, SASP inhibitors (or senomorphics), and improving immune system function [23] and autophagy have been reported [24]. Among them, we were interested in the inhibition of senescence by reducing SASP secretions by ITE treatment, which is a method of modulating senescence-related signaling networks (senomorphics) to attenuate the SASP through autophagy including the regulation of mTOR and NF-κB [6].

**Figure 6:**
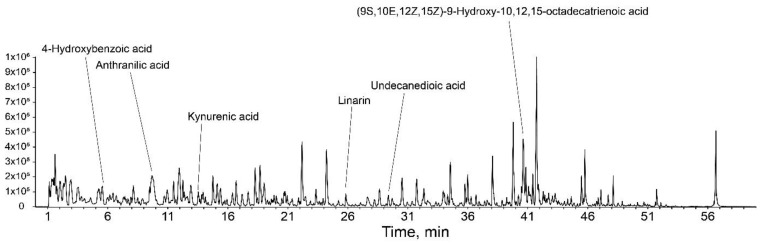
Base peak chromatogram (BPC) of the total ion chromatogram (TIC) of ITE.

Among its physiological effects, *Isatis tinctoria* L. is known to have anti-inflammatory effects including inhibiting TNF-α and IL-1β cytokines [25]. In profiling analysis conducted on the LC-MS/MS system, compounds such as 4-hydroxybenzoic acid, anthranilic acid, kynurenic acid, and linarin were detected (Figure 6). 4-Hydroxybenzoic acid and linarin have been reported to inhibit SASP secretions including TNF-α and IL-6 [26,27], and anthranilic acid and kynurenic acid have anti-oxidant effects [28,29]. However, the mechanism for the anti-inflammatory effect of *Isatis tinctoria* L. has not been elucidated, especially in the replicative senescence model. In this study, we showed that ITE decreased the major marker of senescence, SA-β-gal, the cellular responses to stress (*p53*, *p21^CDKN1A^*, and *p16^INK4a^*), and SASP secretions (Figure 1, Figure 2 and Figure 3). Senomorphics that affect the cellular environment by regulating the SASP through the mTOR inhibitory signals have also been identified [6,30]. The Western blot analysis in this study showed increased mTOR expression in RS-HDFs compared to young cells, but ITE treatment decreased mTOR expression and thereby, the phosphorylation of downstream signals such as S6 ribosomal protein (Figure 5A). In addition, MAPK (JNK and ERK) and IκB phosphorylation were significantly increased in RS-HDFs, indicating an increase in the activated form. However, phospho-MAPK and phospho-IκB were significantly reduced by ITE treatment compared to control RS-HDFs (Figure 4A). Moreover, ITE showed senomorphic efficacy through NF-κB regulation. NF-kB is translocated to the nucleus during cellular senescence and reduced by ITE treatment. These results showed that EGCG and ITE regulated the inflammatory response in RS-HDFs through the NF-κB pathway (Figure 4B). Also, the anti-aging effect of ITE, like rapamycin, was expected to be due to autophagy because these effects disappeared with 3MA treatment, an inhibitor of phosphatidylinositol 3-kinase (PI3K). PI3K plays an essential role in many signaling pathways, including controlling the activation of mTOR, a key regulator of autophagy (Figure 5B) [31]. Previous studies have suggested that SIRT1 negatively controlled mTOR [32], and the protective effect of the specific activator of SIRT1 on HDF senescence was regulated by the autophagy/inflammatory cytokine/cellular senescence pathway [21]. We performed Western blot analysis, which showed that SIRT1 expression was decreased due to degraded cellular metabolism in RS-HDFs compared to young HDFs and increased with ITE treatment (Figure 5A). It has been reported that the proven natural SIRT1 activators were able to modulate and interact with the mTOR pathway [14].

## 5. Conclusions

**Figure 7:**
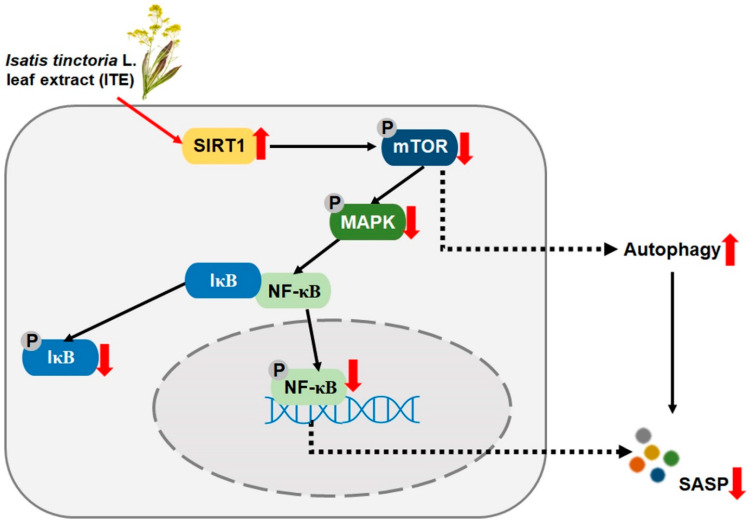
The role of ITE in senomorphic effects through the SIRT1/mTOR/MAPK/NF-κB signaling pathway in RS-HDFs.

Altogether, these results showed the potential for the senotherapeutic agent ITE via the SIRT1/mTOR/MAPK/NF-κB signaling pathway. As shown in Figure 7, ITE induced autophagy and blocked MAPK phosphorylation and nuclear translocation of NF-κB by decreasing phosphorylation of mTOR through upregulation of SIRT1 expression. Through this, ITE modulated senescence-related indicators including SA-β-gal activity and SASP secretions, which resulted in the recovery of cellular function in the replicative senescent model. Therefore, ITE can be suggested as a substance that restores skin by ameliorating cellular senescence.

## Figures and Tables

**Table 1:** Sequences of primers used real-time PCR.

Gene Name	Forward Primer	Reverse Primer
*p16^INK4A^*	CTCGTGCTGATGCTACTGAGGA	GGTCGGCGCAGTTGGGCTCC
*p21^CDKN1A^*	AGGTGGACCTGGAGACTCTCAG	TCCTCTTGGAGAAGATCAGCCG
*p53*	CCTCAGCATCTTATCCGAGTGG	TGGATGGTGGTACAGTCAGAGC
*GAPDH*	CATCAAGAAGGTGGTGAAGCAGG	AGTGGTCGTTGAGGGCAATGC

## Data Availability

Data will be made available on request.
